# Wafer-Scale Particle Assembly in Connected and Isolated Micromachined Pockets via PDMS Rubbing

**DOI:** 10.1021/acs.langmuir.2c00593

**Published:** 2022-05-26

**Authors:** Sandrien Verloy, Bert Vankeerberghen, Ignaas S. M. Jimidar, Han Gardeniers, Gert Desmet

**Affiliations:** † Department of Chemical Engineering CHIS, Vrije Universiteit Brussel, Brussels 1050, Belgium; ‡ Mesoscale Chemical Systems, University of Twente, Enschede 7522 NB, The Netherlands

## Abstract

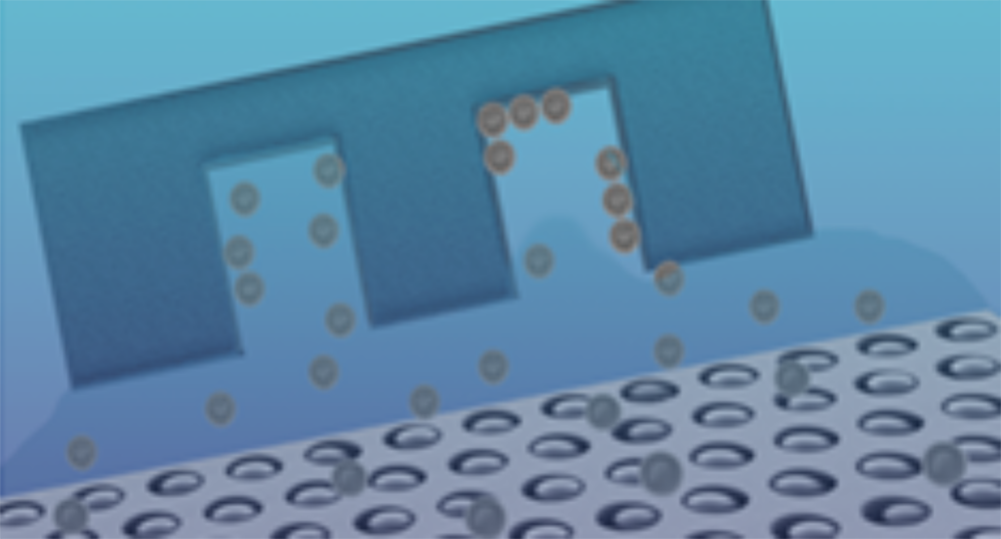

The present contribution reports on a study aiming to find the most suitable rubbing method for filling arrays of separated and interconnected micromachined pockets with individual microspheres on rigid, uncoated silicon substrates without breaking the particles or damaging the substrate. The explored dry rubbing methods generally yielded unsatisfactory results, marked by very large percentages of empty pockets and misplaced particles. On the other hand, the combination of wet rubbing with a patterned rubbing tool provided excellent results (typically <1% of empty pockets and <5% of misplaced particles). The wet method also did not leave any damage marks on the silicon substrate or the particles. When the pockets were aligned in linear grooves, markedly the best results were obtained when the ridge pattern of the rubbing tool was moved under a 45° angle with respect to the direction of the grooves. The method was tested for both silica and polystyrene particles. The proposed assembly method can be used in the production of medical devices, antireflective coatings, and microfluidic devices with applications in chemical analysis and/or catalysis.

## Introduction

The large-scale assembly of spherical particles into a closely packed, hexagonal monolayer or in any other desired two-dimensional (2D) configurations has been of particular interest in many scientific studies. Manufacturing such controlled configurations of micro- or nanoparticles paves the way to a wide range of possible applications.^1^ Particle arrays can, for example, be used in microengineering as a lithographic mask, allowing for the fabrication over a large area of micro- and nanostructures, such as three-dimensional inverse woodpile photonic crystals.^2,3^ Next to this, the periodicity in the monolayers is an essential key feature in a vast number of applications, such as photonics, light manipulation devices, optical and biological sensors, wearable medical devices, chemical catalysis, superhydrophilic, superhydrophobic, or self-cleaning antireflective surfaces, and many more.^1,4−6^ Finding generic particle assembly methods that are independent of the size and the material of the particles is hence of paramount importance. Over the past few decades, various techniques have been developed to find a reproducible and cheap way to assemble particles on a large scale in monolayers on flat substrates or in structured arrays on patterned substrates.^7−10^ Most studies exploit wet assembly techniques, such as Langmuir–Blodgett,^3^ evaporative slope self-assembly,^11^ dip-coating,^12^ drop-casting,^13,14^ spin-coating,^15^ techniques involving the application of an electric field,^16,17^ and many more.^18−20^ Recent research has shown that the surface wettability has a significant effect on the self-assembly of spherical particles from colloidal suspensions upon evaporation.^21^ Alternatively, a large-scale dry particle assembly process has been proposed by the Jeong group.^1,8^ This was successfully applied for the formation of large-area colloidal monolayers on flat, curved, and prepatterned substrates by means of unidirectional rubbing of dry powder using an elastomeric material. Nevertheless, wet assembly methods are in studies still generally preferred over dry methods as the interaction forces between particles in suspensions are noticeably weaker than in a dry state.^22,23^

**Figure 1:**
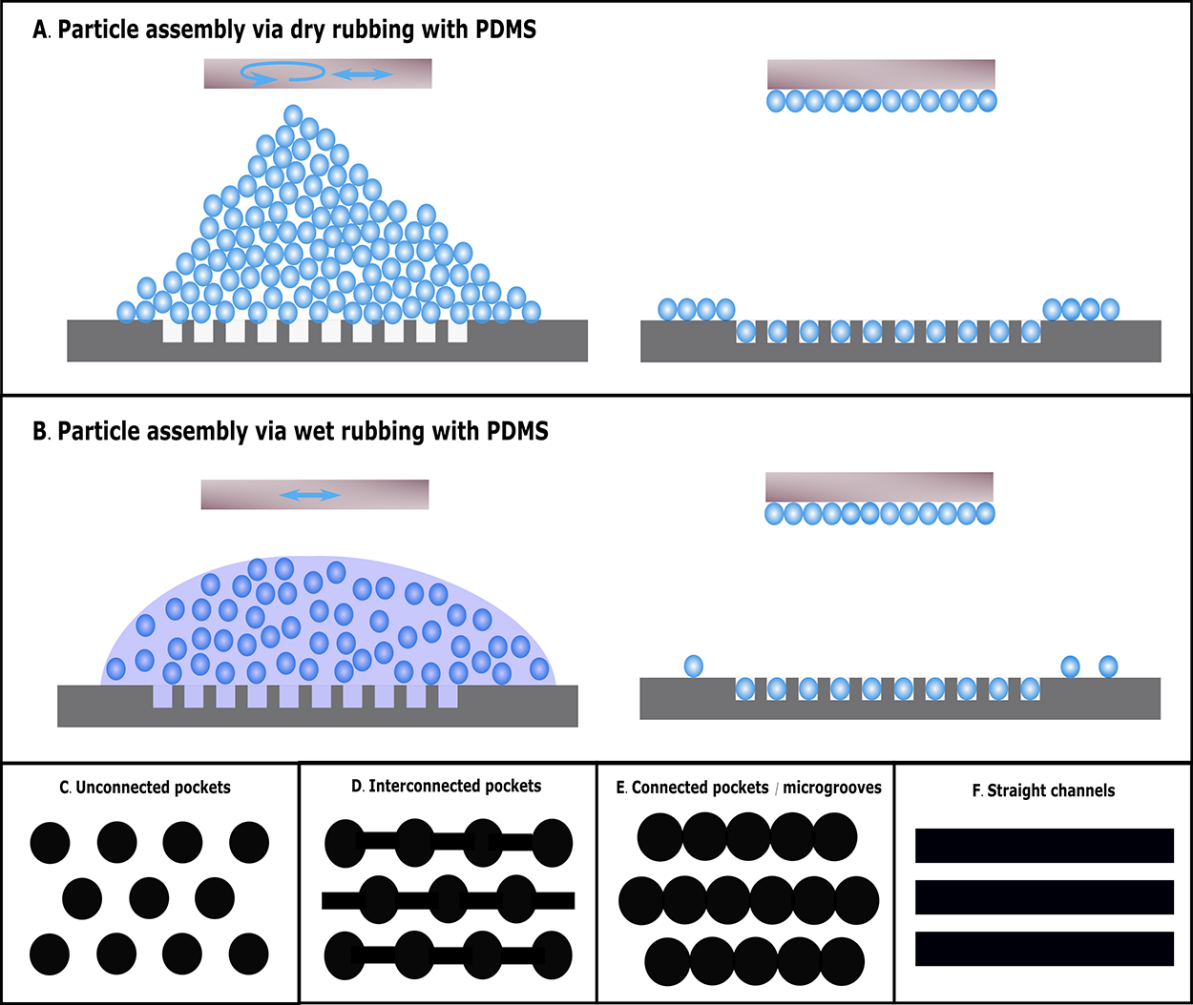
Schematic representation of the adopted dry (A) and wet (B) rubbing procedures for the assembly of particles into micromachined pockets and grooves. Rubbing is carried out with a PDMS tool (brown). The arrows indicate the motion of the rubbing tool: circular for the dry conditions and unidirectional for the wet conditions. (C–F) Schematic top views of the considered pocket and groove structures: unconnected (C), interconnected by channels (D), directly connected to form a microgroove pattern (E), or straight channels (F).

Whereas the aforementioned studies mainly focused on the production of particle arrays and layers on flat or weakly structured surfaces, the present study focuses on the possibility to assemble microspheres (5 and 10 μm diameter) in arrays of micromachined pockets wherein the particles can be completely sunk into the substrate (depth of the pocket ≥ diameter of the particle). Particle assembly is achieved using PDMS rubbing. Dry rubbing (Figure 1A), as introduced by the Jeong group,^1,24^ is used, as well as a novel wet rubbing variant, i.e., by applying the particles in the form of a wet suspension rather than in the dry state (Figure 1B). A comparison between both methods is made. Four different geometries are considered: individually separated (Figure 1C) and linearly connected pockets (Figure 1D,E) as well as straight channel geometries (Figure 1F). The linearly connected pockets, also referred to as microgrooves, are of particular interest as this is a design that allows keeping of the particles in a fixed location while a flow of liquid or gas is sent past them. The other geometries are extreme cases where the geometry of the pockets is varied, either loose pockets or no pockets at all (for straight channels), to test the effect on the assembly process. This form of fully sunk particle entrapment potentially enables the use of stringent cleaning methods (to remove misplaced or excess particles) and allows putting of the particles in a sealed liquid or gas environment such that they can be used in microfluidic applications.^25^ Possible applications are in rapid screening assays,^26^ catalytic microreactors,^27^ or liquid chromatography separations.^28^ Inspired by the work of Izadi et al. on the development of a soft cleaning method for microfabrication,^29^ we also considered the use of patterned elastomeric rubbing substrates as an alternative to the flat PDMS substrates used in most other particle rubbing studies.^1,8,24^ To investigate the versatility of the technique, the developed techniques have been applied for both monodisperse silica and polystyrene microspheres. The focus is on their ability to assemble 5–10 μm particles into arrays of micropockets (either connected or disconnected) designed to receive individual particles (Figure 1C–F).

## Experimental Section

### Materials

The particles were dispersed in solvents (Sigma Aldrich) with ≥99% purity and filtered with a 0.2 μm PTFE syringe filter (Thermo Scientific) to avoid particulate contaminants. Filling of the silicon microgroove patterns with particles was performed under ambient conditions. Experiments were performed with hydrophilic silica particles (diameters of 10.02 ± 0.32 and 4.64 ± 0.14 μm) and hydrophobic polystyrene particles (diameter of 10.14 ± 0.12 μm) that were purchased from Microparticles GmbH. Standard deviations on the particle diameter were supplied by the manufacturer.

### Fabrication of the Patterned Silicon Substrate

Microfabrication processing was performed at the MESA+ Nanotechnology Institute of the University of Twente. A variety of designs of the microstructures were tested and incorporated on a single silicon substrate. The design consisted of freestanding circular pockets with varying distances in between (the diameter was varied from 10 to 13 μm), connected microgrooves with varying overlaps of adjacent circular pockets, and straight channels. The microstructures were fabricated on 4-inch silicon substrates (one side polished, (100), p-type) by standard lithographic patterning^30^ followed by a deep reactive-ion etching (DRIE) process to transfer the pattern into bulk silicon. A three-step DRIE process, capable of achieving nearly vertical sidewalls with a scallop size of 30 nm, was used.^31^ The process was based on the use of C_4_F_8_ and SF_6_ gases and was implemented on a PlasmaPro 100 Estrelas machine (Oxford Instruments). The pattern was etched 12–13 μm deep into the silicon. Prior to the particle assembly experiments, the substrate was thoroughly cleaned by O_2_ plasma (Tepla 360) to remove fluorocarbon and photoresist residues and finally with HNO_3_ to remove any remaining organic residues.

### Fabrication of Master Molds and the PDMS Rubbing Tool

Both flat and patterned polydimethylsiloxane (PDMS) rubbing tools were used for the particle assembly. To produce the flat rubbing tools, flat PDMS sheets were made by pouring PDMS (SYLGARD 184 silicone elastomer kit; Dow, Inc.) in a Petri dish and cross-linking it in an oven at 60 °C for at least 4 h. After curing, this PDMS slab was cut into the desired size and shaped with a sharp blade, e.g., a scalpel. To produce the patterned PDMS tool, master molds were fabricated on regular 4-inch silicon substrates by patterning in a negative photoresist (MicroChem NANO SU8). The negative image of the desired pattern for the PDMS rubbing tool was transferred to the SU8 layer by means of standard optical lithography. Different patterns were considered, involving both pillars and ridges of different sizes in a range from 10 to 400 μm. The rubbing tool was made by pouring PDMS (silicone elastomer:initiator ratio of 10:1) on top of the master mold substrate and cross-linking it in an oven, in the same manner as described above for the flat sheets. The resulting PDMS rubbing tool was obtained by separating PDMS from the master mold and cutting it into the desired size and shape.

### Dry and Wet Particle Assembly through Rubbing

#### Experimental Setup

The particle assembly via rubbing occurred very similar for the dry and wet procedures: the silicon substrate was secured, and particles were supplied to this substrate before rubbing the flat or patterned PDMS piece manually with either a circular or a back-and-forth movement. The number of strokes performed was in most cases in the range between 10 and 20. For extra support and to ensure a uniform contact of PDMS with the substrate, the PDMS was taped or glued to a 3D printed plastic holder. All the rubbing experiments were conducted in triplicate.

#### Dry Methods

A scoop of about 50 mg of silica or polystyrene particle powder was weighed and supplied to a silicon substrate containing micropockets. The dry assembly process depicted in Figure 1A started by performing a circular rubbing motion for about 30 s to a minute (corresponding to 10–20 strokes) with the PDMS rubbing tool on top of the silicon substrate, and this was defined as a single rubbing step. Subsequent visual inspection with an optical microscope was used to determine whether additional rubbing steps were needed to achieve the desired filling ratio of 99–100%.

#### Wet Methods

The particles were suspended in a solvent at the desired concentration (standard rubbing solutions were 20 and 50 mg/mL), before thorough mixing with a vortex mixer along with half an hour in an ultrasonic bath at maximum power. A drop of known volume (20–100 μ.L) of the particle suspensions was supplied to the substrate for particle assembly. The drop was left to evaporate for at least 1 min, which was defined as the waiting time, to allow the dispersed particles to sediment toward the underlying substrate. The wet assembly procedure was initiated by placing the PDMS rubbing tool on top of the previously deposited drop. Subsequently, the rubbing motion was initiated, either a unilateral motion or a circular motion depending on the size of the substrate. For full-size wafer substrates, circular motions can be performed (see Movie S1). This was not possible for very small substrates, for which the rubbing movement was only executed unilaterally. The rubbing motion was continued until all the solvent had evaporated or preferably even a little before. A single wet rubbing procedure as defined above could be repeated multiple times until the attained filling ratio (see below) was nearly 100%.

#### Particle Counting Methods

To quantify the quality of the assembly processes, a filling ratio (FR) and an error ratio (ER) were defined for the silicon substrates after rubbing. The filling ratio was defined as the proportion of available pockets that had been filled with particles. The error ratio was defined as the number of particles deposited on unwanted locations divided by the total number of pockets. In between consecutive rubbing experiments, the filling ratio was visually checked with optical microscopy. The FRs and ERs were obtained by examining scanning electron microscopy (SEM) pictures with an image processing program (ImageJ).^32−34^ The FRs and ERs were determined on a subset of smaller areas as calculating the ratios across the entire silicon substrate was a very laborious and time-consuming task due to the required image stitching and the number of particles per sample (depending on the pattern, this was in the range from 190,000 to 360,000 particle pockets per sample). The subareas used to determine the filling and error ratios were at least 300 × 300 μm and contained a minimum of 1000 pockets and were all chosen randomly.

## Results and Discussion

### Dry Rubbing

**Figure 2:**
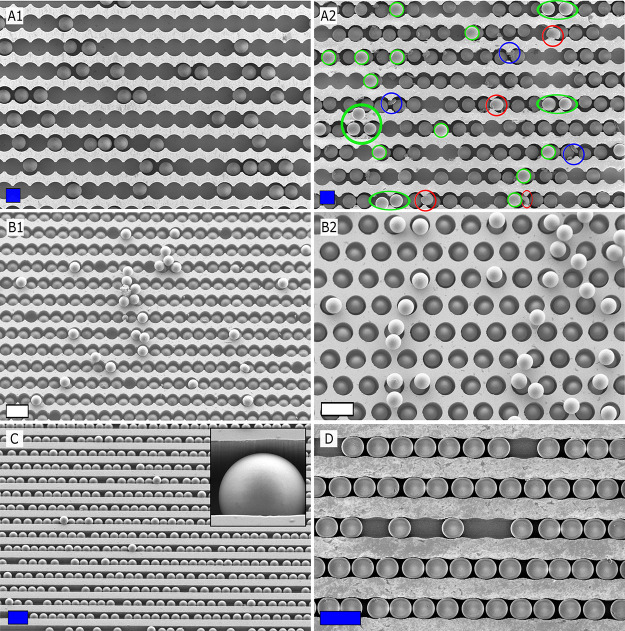
SEM pictures of typical dry assembly experiments carried out with 10 μm silica (A) and 10 μm polystyrene (B) particles. Arrays have either been filled by applying a single (A1,B1) or at least three consecutive (A2,B2) rubbing runs. (C,D) Typical results obtained with 5 μm silica particles in straight and wavy channels. Color code in A2: broken particles (red circles), debris from the rubbing tool (blue circles), and particles forming a second layer or at unwanted locations (green circles). Scale bars: blue = 10 μm and white = 20 μm.

It is well-known that microparticles tend to aggregate due to substantial cohesive interactions, particularly under dry conditions.^35,36^ This highlights the necessity of applying a sufficiently strong shear force during the rubbing motion to separate agglomerates into single particles required to assemble 2D particle arrays.^36^ The initial dry rubbing experiments were performed with a flat PDMS sheet using 10 μm silica or polystyrene particles on silicon chips containing microgroove patterns (Figure 1A). After a single rubbing run, the filling of the microgrooves with silica particles was relatively poor (filling ratio (FR) =20 – 25%), as can be seen in Figure 2A1. Repeating the dry rubbing process at least three times allows attainment of a considerably higher FR (70 – 79%), as can be seen from Figure 2A2. The FR that can be achieved with this repeated procedure is however still far from satisfactory. Especially because it was also found that the FR varies immensely along the substrate, consequently, the reproducibility for larger samples is poor. It is assumed that the relatively high rubbing forces needed to separate the strongly agglomerated microspheres into single particles cannot easily be maintained at a constant level during the rubbing process. This inevitably leads to a maldistribution of the assembly quality.

For polystyrene particles on the other hand, it was found that a single dry rubbing run was sufficient to obtain an FR of 80 – 85%, as is shown in Figure 2B1. In many instances, repeated rubbing with polystyrene particles even led to nearly complete filling (90 – 99%) of the arrays (Figure 2B2). The error ratio (ER) (= the fraction of particles at undesired places) was for both types of particles within a range of 1–10% for a single rubbing experiment but could attain values of up to 50% after consecutive rubbing experiments. The dry rubbing particle assembly for 5 μm silica particles (Figure 2C,D) yielded similar results to those obtained for the 10 μm silica particles.

It is assumed that the large ERs are due to the large particle excess that is needed to start-up the process. In a dry process, one scoop of particles corresponds to about 50 million particles (corresponding to an excess ratio of about 100:1 compared to the number of empty pockets), which are directly scooped onto the substrate. This massive number of particles is needed for a smooth rubbing motion as the particle layer formed between the silicon substrate and the rubbing tool acts as a lubrication layer.

The different behavior of the silica and polystyrene particles can potentially be ascribed to their initial state. When taking a closer look at the surface of the particles (at SEM pictures in Figure S1), it can be concluded that the polystyrene particles have a relatively large surface roughness compared to the silica particles, implying that there are fewer cohesive interactions among the polystyrene particles than among the silica particles. In addition, due to the silica particles’ hydrophilic nature, due to humidity, a capillary force may act between them, leading to a strongly agglomerated state. On the other hand, due to the hydrophobic surface of polystyrene particles, it is expected that a water layer is absent on their surface, and concomitantly, the PS particle surfaces may carry a significant electrical charge, leading to a repulsive Coulombic force among the polystyrene particles with the same polarity.^22,36^ As the rubbing motion is applied on the particles, they can either slide or roll across the substrate, provided that the applied force surpasses the friction force restraining the movement of the particles. Considering that the dry silica powder comprises large and densely packed aggregates at the start of the rubbing process, it can be intuitively argued that they will slide in one piece across the substrate.^36^ A sufficiently strong force is required to separate the agglomerates into individual particles before they can get trapped inside the micropockets. The polystyrene particles, on the other hand, are mostly present as single particles, except for a few small aggregates. Consequently, the external force needed for the PS particles to move across the substrates is smaller than for the silica particles.^36^ As such, the polystyrene particles can be more easily assembled within the microgroove patterns.

The dry rubbing experiments have been carried out on a variety of geometrical patterns. The filling results are very similar on geometries where the pockets are interconnected (Figure 2B1) as well as where they are not connected (Figure 2B2). Interestingly, when using straight channels (Figure 2C) or channels with a mild wavy pattern (Figure 2D), the filling process appeared to go much smoother for silica particles, and significantly higher filling ratios were observed (roughly 20% for isolated or partially connected pockets versus roughly 70% for straight or wavy channels). Our hypothesis is that the assembly of the particles in the microgrooves is hindered by the sharp edges that are a result of the overlapping circular pockets. We also suspect that these sharp edges might also explain the increased particle breakage that we observed for these samples.

It should be noted that although the filling ratios obtained for dry rubbing polystyrene could in some cases attain nearly 100%, there was always some local variation in the number of unwanted excess particles (0 – 10%) deposited on the side of the pockets. Some minor defects, such as a second layer or slightly misplaced particles (Figure 2A2), are quite commonly observed phenomena in the case of dry rubbing, regardless of the pocket or channel geometry. Although dry particle assembly methods have been successfully employed in the past to achieve assembled arrays comprising colloidal polystyrene particles on 2 × 2 cm^2^ stretchable substrates^8^ as well as on rigid 10 × 10 cm^2^ PDMS-coated silicon substrates,^24^ it proved difficult for us to obtain perfect assemblies on the wafer scale, especially in the case of the silica particles (Figure 2A2). One explanation could be that we employed very rigid silicon substrates, while the Jeong group^1^ mentioned that the template flexibility possibly aids to alleviate some of the sterically induced elastic stress on particles in a closely packed structure. Another reason for the difference in packing quality between the results obtained here on rigid silicon substrates and the other studies^1,8^ where only soft elastomeric substrates are involved is the manner in which particles move across these substrates. For rigid substrates, the particles tend to slide more, while for elastomeric substrates, particles often display a perfect rolling motion. The latter significantly enhances the packing efficiency in the case of elastomeric (coated) substrates.^1^

To improve the quality of the dry rubbing method, we made use of an idea mentioned in a study of Izadi et al.^29^ who proposed to use patterned instead of flat PDMS sheets for the dry removal of contaminating particles during microfabrication processes. They showed that the use of an elastomer cleaning tool that is patterned with pillars or ridges can effectively remove particles without damage to the underlying surface. The main advantage of the patterned surface is that the attracted particles get transported away from the contact interface, along the sidewalls of the patterned structures. This prevents the removed particles from recontaminating the substrate. Inspired by this work, we investigated whether the use of a patterned rubbing tool would lead to higher-quality rubbing assembly results than those obtained with flat rubbing surfaces. For this purpose, patterned PDMS sheets marked by either rectangular bars or a pillar array geometry have been tested. The key idea behind the use of the patterned PDMS sheet is that this approach would allow the combination of the particle assembly and the removal of the excess particles in a single step. However, the results obtained with a patterned PDMS rubbing tool are in the case of dry assembly rather poor, with FRs not higher than 20%. Inspecting the patterned PDMS rubbing tools after rubbing revealed that the upper parts of the patterned structures, i.e., the parts that came in direct contact with the pocket substrate, were covered by partial monolayers, while the regions in between the ridges were fully loaded with silica particles. These particles were no longer available to fill the pockets on the silicon substrate. The patterning of the PDMS rubbing tool also led to a distortion of the tribological behavior of the rubbing tool. The presence of the ridges reduced the contact area between PDMS and silicon and prevented the formation of a lubrication layer covering the entire surface. This appeared to hinder the movement of the particles, which no longer moved swiftly over the silicon wafer surface and remained very close to the point where they first made contact with the patterned rubbing tool, making less particles available for the assembly.

Collectively, these results show that dry assembly performed with either a patterned or nonpatterned PDMS sheet gives poor results. Typical FRs after the first rubbing procedure are in a wide range between 50 and 90% (depending on the geometry of the pocket array), and the typical percentage of misplaced particles is on the order of 20–30%. Repeating the rubbing process increases the FR but at the same time also leads to an increase in the number of excess particles (either present as a second layer of particles resting on top of the particles inside the pockets or next to them (see green circles in Figure 2A2)). In addition, it also increases the contamination by PDMS debris detaching from the rubbing tool during the rubbing (see blue circles in Figure 2A2). Finally, the dry method requires a vast overload of powder particles (excess ratio of 100:1), rendering it a very inefficient process. Next to that, the dry rubbing motion increases the probability for particles to break (see red circles in Figure 2A2).

### Wet Rubbing

Given the observed limitations of the dry processes and considering that the strong interaction forces, e.g., the capillary forces, van der Waals forces, and tribocharging-induced electrostatic forces, can be alleviated under wet conditions, we moved to testing the pocket and groove-filling process under wet conditions. One advantage of the wet method is that the solvent separates the particles from each other by the presence of a solvation shell around the particles, which will be addressed at a later stage in this report. In addition, the van der Waals force is reduced, and concomitantly, the electrostatic force reduces, as the solvent will allow for an easy and fast dissipation of rubbing-induced tribocharges on the particle’s surface. A particular advantage for the present purposes is that the solvent lubricates the surface of both the silicon substrate and the particles, thus significantly reducing the friction force between the particles and the two substrates. Especially for the case of the pockets, less force will be required to push the particles inside the pockets due to the lubrication layer, and the risk of the particles getting stuck halfway inside the pocket by capillary or other forces is diminished by the presence of this liquid layer. Consequently, the particles can move more freely over the rubbing substrate than in a dry state, thus minimizing the risk of particle breakage or damage to the silicon support structure.

**Figure 3:**
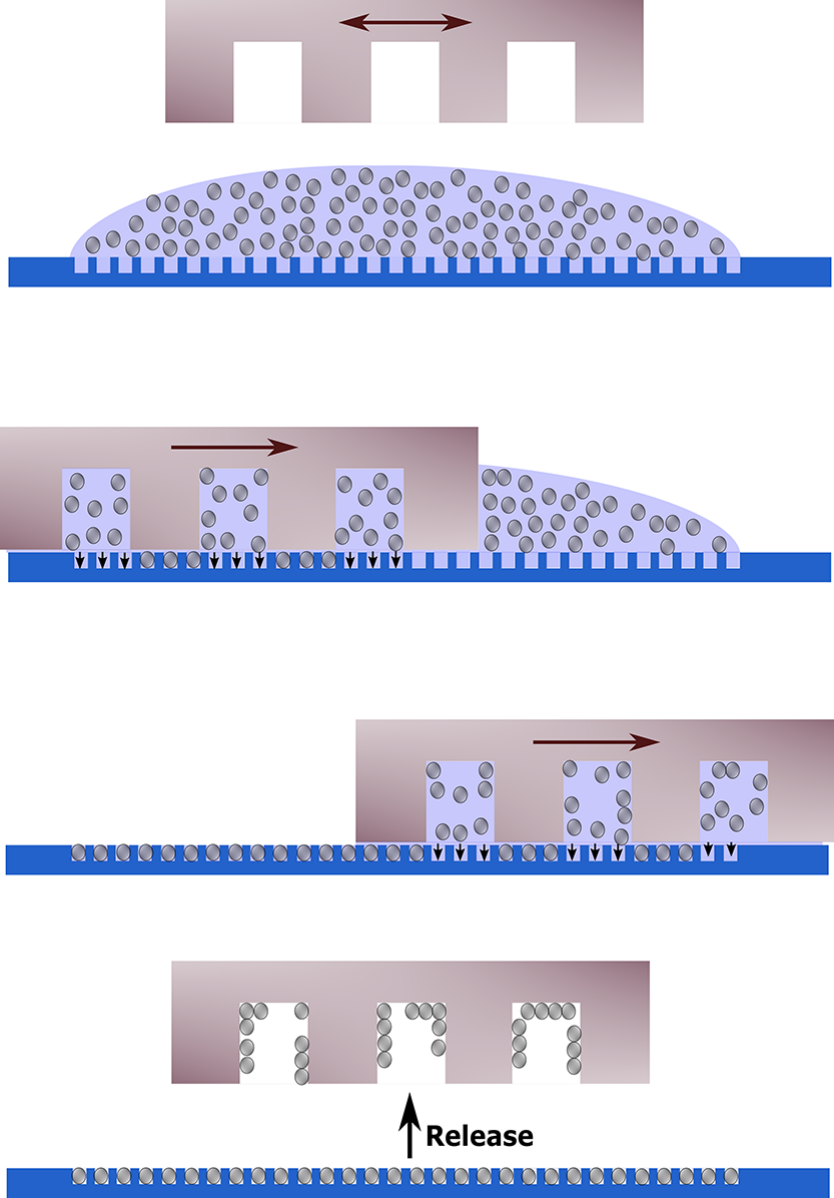
Schematic overview of particle (gray) assembly via wet rubbing with a patterned PDMS rubbing tool (light brown) on a silicon substrate with microgrooves (blue).

**Figure 4:**
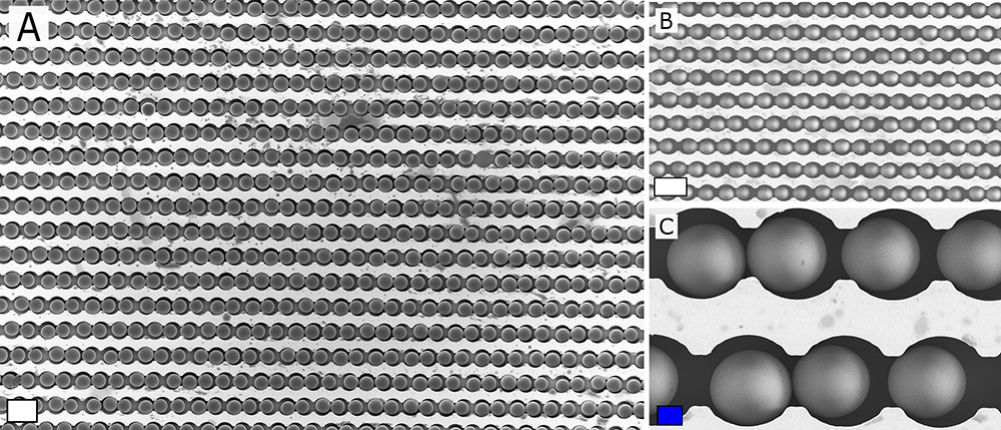
SEM pictures at different scales of typical (A–C) wet assembly experiments carried out with 10 μm silica particles by means of a single wet rubbing run with a patterned PDMS rubbing tool under optimal conditions. Sample dimensions: 10 × 10 mm. Experimental conditions: applied amount of particles = 30 μL of a 20 mg/mL suspension in ethanol; 1 min evaporation time before initiating a circular rubbing motion with a patterned PDMS substrate with 50 μm grooves. Scale bars: blue = 10 μm and white = 20 μm.

**Figure 5:**
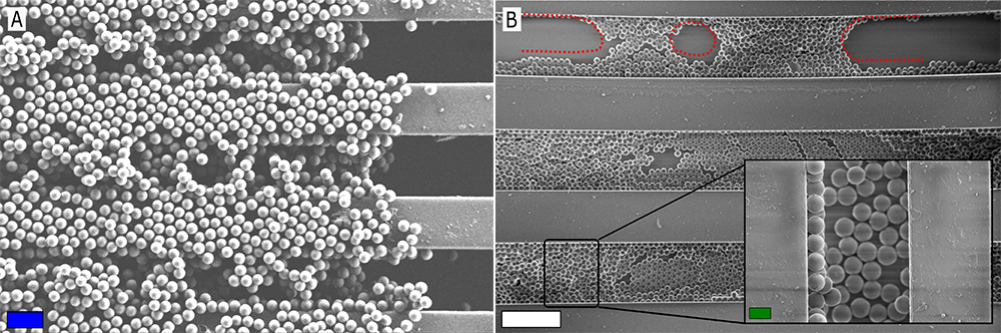
SEM pictures of the patterned PDMS rubbing tool after dry (A) and wet (B) rubbing assembly processes with 10 μm silica particles. The ridge pattern on the tool is 50 μm wide as well as deep. Evaporative fronts are indicated in red. The quasi monolayers that have been formed on the sidewalls and bottom of the grooves in the PDMS rubbing tool can be seen in the right bottom corner of (A). Scale bars: blue = 30 μm, green = 10 μm, and white = 100 μm.

All wet assembly experiments started by supplying the particles through a liquid suspension on the (patterned) substrate (Figure 1B) followed by a short evaporation time before initiation of the rubbing motion. Using wet rubbing (Figure 3), the filling ratio observed for the silica particles after a single wet rubbing experiment was in most cases nearly perfect and consistently reached 99% or higher combined with an ER below 1% (Figure 4), which is in strong contrast with the dry process where the filling ratio obtained after one rubbing iteration was approximately 20%. To ensure that the observed high filling ratios are not a merely a result of an evaporation-driven assembly process and that the rubbing motion is essential, a single drop of the particle suspension was supplied to the silicon substrate and left to dry without rubbing. Inspection with SEM revealed that some pockets were filled with particles, although not nearly as much as for the case where the silicon substrate was rubbed with the PDMS tool. The results show that the rubbing motion indeed aids the particle assembly into the micropocket array (Figure S2). The wet rubbing process also differs from the dry process in the way that the particles are supplied to the substrate. In the wet assembly process, the particles are supplied by transferring a small volume (30–100 μL) of suspended particles with a micropipette to the pocket regions of the substrate. Under wet conditions, considerably fewer particles need to be supplied than under dry conditions, where a high excess is needed for lubrication purposes. By controlling the concentration, the number of particles supplied to the silicon substrate was typically around 4–10 million, corresponding to an excess ratio of 3:1 to 9:1, i.e., considerably less than that in the dry method (100:1). Furthermore, the micropipette-dispensed particles are more evenly distributed at the start of the rubbing process. Additionally, as the solvent strongly promotes the breakup of the agglomerates, considerably less rubbing force is needed compared to the dry rubbing process where the agglomerates first need to be separated into single particles to be able to position themselves in the pockets on the silicon substrate. It is also easier to maintain the smaller required rubbing force uniform over the entire silicon wafer, leading to a much more evenly distributed assembly quality. A closer inspection of the used patterned PDMS rubbing sheet reveals that the rubbing tool looks quite different after a wet rubbing process compared to a dry rubbing process as there are noticeably fewer particles present and they are in monolayer formation (Figure 5). The straightforward explanation for this observation is the fact that considerably fewer particles were supplied under wet conditions. As the PDMS tool is no longer overloaded with particles, the number of excess particles that were deposited on unwanted locations decreased significantly. The PDMS tools that were patterned with circular pillars did not yield adequate results as often, the substrate was left with stripes of particles, a direct consequence of the fact that the pillar pattern does not have enough vertical surface area that can lead the excess particles away from the silicon wafer.

Subsequently, several parameters of the wet rubbing process were varied to find the optimal conditions. It is possible that for geometries other than those presently studied, the optimal parameters might slightly differ from what is discussed here, but in general, we have found that the selected conditions work for a variety of patterns and particles. Since the success of the wet assembly technique depends highly on solvent parameters such as the evaporation rate and polarity of the solvent, several solvents were tested (Figure S3). Especially, polar solvents with a high evaporation rate like ethanol and isopropanol seem to be very suitable for the wet assembly procedure (Figure S3A–D). It should be noted that ethanol yields a more uniform filling over large-scale areas when compared to isopropanol, most likely because of the very high evaporation rate of isopropanol. Acetone was dismissed as it is well-known for leaving traces upon evaporation (Figure S3E,F). Other solvents with a reasonably low vapor pressure, e.g., 1-hexanol and water, were found to be less suitable (Figure S3G,H). The solvent used should have a weak to very polar character to facilitate the suspension for polar particles in a solvent, such as silica or sulfonated polystyrene particles. Due to the polar nature of the solvent molecules, they can position themselves in a stabilizing configuration around the particles, forming what is referred to as a solvation sheath or shell. This solvation shell will stabilize single particles to stay in suspension by electrostatic repulsion (see the DLVO theory) and prevent the formation of aggregates by creating a distance between the particles. Further, we noticed that it is highly desirable that the solvent evaporates quickly enough as we want the final state to be dry. On the other hand, the evaporation rate should not be too fast as this leaves the experimenter with insufficient time to ensure an adequately uniform rubbing motion over the entire silicon substrate. Another reason why solvents with a low evaporation rate are unfavorable is the manner with which the PDMS rubbing tool is lifted at the end of the rubbing procedure, as this motion needs to be swift and the remaining liquid layers of the solvent could possibly contain particles that will ultimately be left on unwanted locations after evaporation. As the solvation of the particles weakens and eventually vanishes upon solvent evaporation, the particles become more likely to stick to the PDMS rubbing substrate than they are to enter the pockets.

Experiments were also performed to assess the effect of the particle suspension concentration as well as of the timing of the different steps of the wet rubbing process. In previous experiments, the particle suspensions were allowed to settle for approximately a minute before rubbing. This appeared to be highly critical because the obtained filling ratios did not reach values higher than 40% without allowing for this waiting time, i.e., when the rubbing motion was initiated immediately after depositing the suspension in ethanol (Figure S4). This was probably because the particles inside the deposited drop did not get the chance to sediment to the bottom of the liquid layer, i.e., toward the patterned silicon substrate. When starting the rubbing motion immediately, most of the particles in the suspension get swept off to the sides of the silicon substrate without having a chance to come in contact with the silicon substrate. By using Stokes’ law,^37^ the time it takes for 10 μm silica particles to sediment to the bottom of a 40 μL droplet on a 1 × 1 cm^2^ sample was estimated to be around 40 s. Thus, it can be concluded that waiting times of approximately 1 min allow ample time for sedimentation of the particles and accordingly yielded a more satisfactory filling ratio (90% and higher).

It was found that the most crucial aspect of the method was keeping enough liquid present during the whole rubbing procedure. The presence of a solvation layer around the particles throughout the procedure will provide sufficient lubrication to reduce friction as well as an easier dissipation of charges consequently reducing electrostatic forces. Slightly better results were obtained for longer waiting times, up until 4 min in the case of the presently considered silicon substrates (volume of 100 μL spread out along a sample of 0.5 × 50 mm). Much longer waiting times resulted in insufficient lubrication for rubbing as the parts of the wafer already started to dry up and particles became more prone to being picked up by the rubbing tool. For this reason, it is essential to stop the rubbing motion shortly before all the solvent has evaporated in order to achieve a high FR. This leads to the conclusion that a trade-off exists between starting the rubbing process soon enough to maintain sufficient lubrication and waiting long enough for the particles to settle toward the microgroove substrate. These observations could possibly be explained by thin-film entrainment, first described when studying particle assembly by dip-coating.^38^ This regime occurs at a critical withdrawal speed during the dip-coating process, i.e., when the thickness of the liquid film is about the same as the particle diameter. For the present purposes, where it is attempted to deposit the particles inside the pockets or microgrooves with as little as possible particles or aggregates on the top or on the side, this theory suggests that the optimal liquid film thickness can be expected to be of the same order as the particle diameter. It can be expected that analogous to the simulations made for the dip-coating process,^38^ the particles would be forced to assemble into a structured configuration inside the liquid layer. If the liquid layer is much thicker than the particle diameter, then drag forces (and other forces that are potentially also active) can still move particles within this liquid layer on top of each other, thus risking the deposition of a second layer and/or aggregates on the substrate.

**Figure 6:**
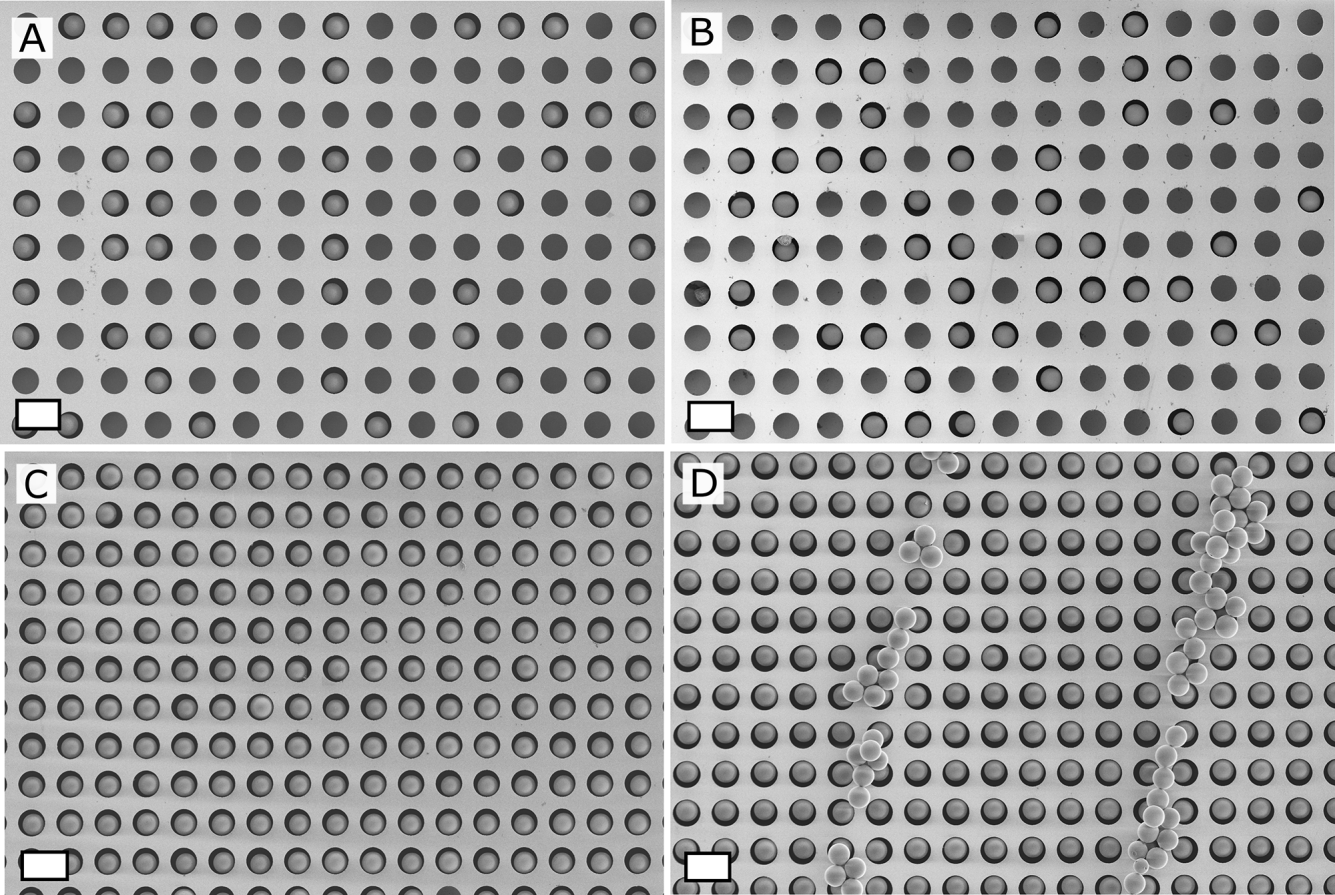
SEM pictures showing the effect of a suspension concentration variation study (wet assembly): 5 (A), 10 (B), 20 (C), and 50 mg/mL (D). Other conditions: deposition of a 30 μL drop of a suspension in ethanol to the substrate; 1 min evaporation time before initiating a unidirectional rubbing motion with a patterned PDMS substrate with 50 μm ridges. Scale bar: white =20 μm.

After deposition of the droplet, the concentration inside the droplet increases as evaporation occurs throughout the rubbing procedure. The influence on the particle assembly of the initial concentration of the particle suspensions was thus investigated. Different concentrations, ranging from 5 to 50 mg/mL, for the suspensions were tested (Figure 6). The best results (FR = 100%, ER <1%) were obtained for a 20 mg/mL concentration of a drop of 60 μL, which corresponds to an excess ratio of about 3:1. For lower concentrations, filling ratios became inadequate (around 30%) and very nonuniformly distributed. In the experiments conducted at a higher concentration (50 mg/mL corresponding to an excess ratio of about 7:1), the FR obtained was 100%, but many extra particles and even aggregates were deposited on top of or next to the pockets (ER = 1–10%). The presence of these extra particles suggests that this excess ratio is too high, and the patterned PDMS rubbing substrate can no longer remove all excess particles, which are then left on random locations on the micropocket substrate. The thin-film entrainment concept also provides a possible explanation for the increased number of unwanted particles present for longer evaporation times (Figure S4). As the solvent in the particle suspension evaporates over a certain time, some areas of the silicon substrate will start to become dry, and particles or aggregates present in these areas will no longer be suspended in the solvent, resulting in the deposition of unwanted particles on the silicon substrate.

**Figure 7:**
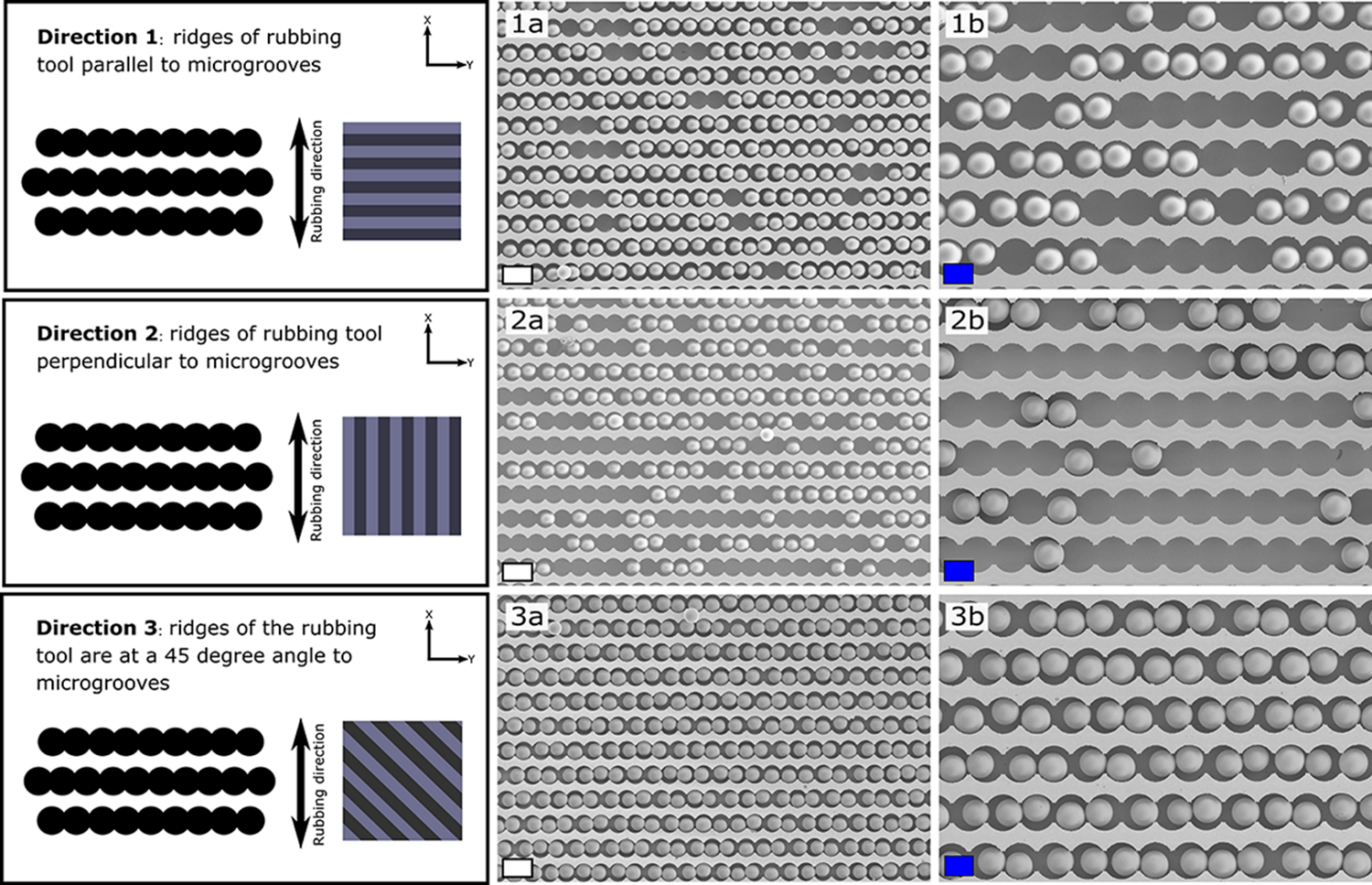
Effect of the rubbing and PDMS pattern orientation on the achieved filling quality (wet assembly): ridges of the rubbing tool parallel to the microgrooves on the substrate (1a,b); ridges of the rubbing tool perpendicular to the microgrooves on the substrate (2a,b); ridges of the rubbing tool under a 45° angle with respect to the microgrooves on the substrate (3a,b). Scale bars: blue = 10 μm and white = 20 μm.

We also investigated the design of the line pattern on the PDMS rubbing tool. This pattern can be viewed as a set of small parallel microblades, similar to a doctor blade coating setup, albeit that here, the “blades are made from an elastomeric material”.^39^ More specifically, we investigated the effect of the orientation of the ridge pattern on the PDMS tool with respect to the main directions in the microgroove arrays where the pockets are connected linearly (Figure 7). It was found that the filling occurred in a preferential direction in these geometries (Figure 7, directions 1 and 2). When the ridges of the PDMS tool are oriented perpendicular or parallel to the main axis (*y*), the ridges will be parallel to the pockets along the *x*-axis and *y*-axis, respectively. The FRs achieved for direction 1 were around 90%. In this scenario (Figure 7, direction 1), where the PDMS tool is repeatedly moved in the axial direction along the whole microgroove pattern, it is plausible that due to the elastic properties of the PDMS material, particles get easily picked up again by the PDMS tool even when these particles are already residing in a pocket. This removal of particles by the patterned PDMS tool is highly dependent on the pressure that is applied manually during rubbing, showing the importance of finding an automated and hence more reproducible way of executing the rubbing process. This becomes imperative when (almost) all the liquid has evaporated. As long as some liquid remains present, capillary bridges can be formed between the particle and the sidewalls of the pockets, anchoring the particle in the pockets through capillary bridge formation. If all the liquid has evaporated, then the particles can get picked up again by the rubbing tool. Almost instantly after contact between the PDMS rubbing tool and the deposited particle suspension on the silicon substrate, the space between the ridges on the PDMS is filled with the particle suspension by capillary action. In the case where the ridges of the rubbing substrate are oriented parallel to the main direction of the column (Figure 7, direction 2), the axial direction of the rubbing process leads to a situation wherein some regions along the main axis are in constant contact with the PDMS ridge alternated with regions in contact with the particle suspension inside the groove. FRs achieved for direction 2 were around 90%. The parts of the silicon substrate that are in constant intimate contact with the ridges of the PDMS have no means of supplying extra particles to the pockets underneath, while on the other hand, some regions are in constant contact with the particle suspension, and excess particles will be deposited there due to evaporative behavior of the suspension. The 45° method (Figure 7, direction 3) seems to provide an ideal balance between intimate contact of the ridges with the underlying substrate and a steady supply of particles from the suspension in the ridges to the underlying substrate to fill up the remaining empty pockets. This method gave noticeably better results (>99% filling in nearly all cases) than for any other orientation of the ridges in the PDMS with respect to the column (Figure 7).

To investigate the effect of wetting properties of the substrates on the assembly process, both the hydrophilicity of the rubbing substrate and that of the PDMS rubbing tool were altered. A very thin layer of fluorocarbon was deposited conformally on the patterned silicon substrate,^140^ and it was found that after wet rubbing, the FRs were as equally good as for the uncoated substrates. Initially, when depositing the drop of the particle suspension on the hydrophobic silicon substrate, it will not spread out as easily, but rubbing with the patterned PDMS tool will ensure that the suspension is spread out over the entire substrate during rubbing. Increasing the hydrophilicity of the PDMS rubbing tool did not result in any change in the outcome of the rubbing experiments. The size of the ridges of the rubbing tool was varied, and it was determined that a width about 5 times as large as the particle diameter is sufficient to have enough space in the tool to capture the excess particles. Larger widths of the ridges lead to the increased bending of the PDMS tool and yield slightly worse FRs due to nonuniform contact with the underlying substrate during rubbing.

It was also observed that both the size and the depth of the pockets are crucial parameters. Obviously, the fit of the pockets should not be too tight as slightly oversized particles can get stuck before reaching the bottom of the pockets. On the other hand, if the diameter of the pockets is too large, then multiple particles can enter a single pocket, which in turn can lead to excess particles adhering to the primary trapped particle. Optimal size ranges were found to be 1.1–1.3 times the particle diameter for both the depth and the diameter of the pockets. Particle assembly yielded good FRs (≈99%) when the depth of the pockets was somewhere in the range from 0.5 to 1.3 times the particle diameter for a single layer, and lower depths smaller than the particle radius *R* resulted in bad FRs (<60%) due to the particles being able to roll out of the pocket again during the rubbing motion (Figure S5A–C). If desired for the application, then even multiple layers of particles can be assembled within the microgrooves or pockets (Figure S5D).

**Figure 8:**
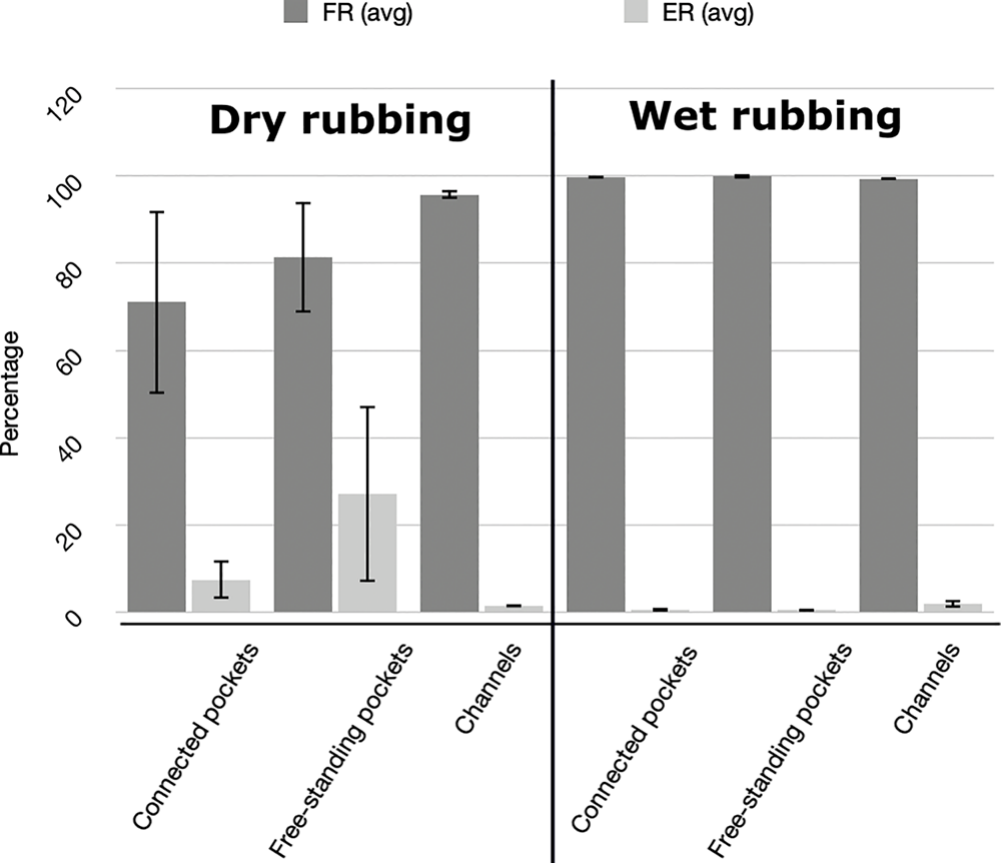
Graphs showing the average values for the FR and ER after dry (left) and wet (rubbing) experiments for varying geometries of the patterned silicon substrate (connected pockets, freestanding pockets, and channels) filled with 10 μm silica particles. Experimental conditions for dry rubbing (left): deposition of a 10 mg scoop of dry particles to the substrate followed by unidirectional or circular rubbing motion with a flat PDMS rubbing tool; for wet rubbing (right): deposition of a 30 μL drop of a 20 mg/mL suspension in ethanol to the substrate; 1 min evaporation time before initiating a unidirectional rubbing motion with a patterned PDMS substrate with 50 μm ridges.

**Figure 9:**
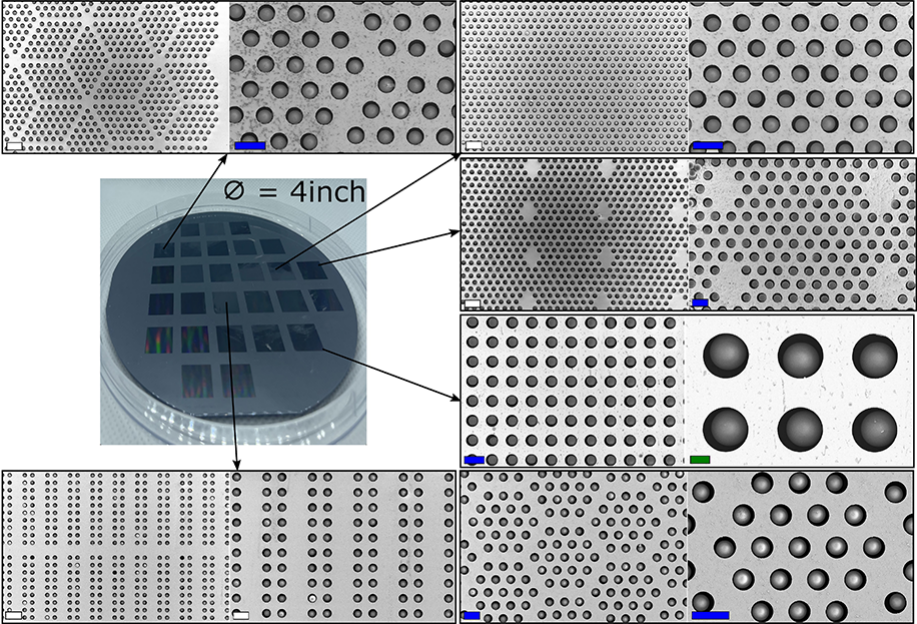
SEM pictures of particle assembly on substrates with different geometries (wet assembly, 10 μm silica particles). Experimental conditions: deposition of a 30 μL drop of a 20 mg/mL suspension in ethanol to the substrate; 1 min evaporation time before initiating a unidirectional rubbing motion with a patterned PDMS substrate with 50 μm ridges. Scale bars: blue = 20 μm, green = μm, and white = 40 μm.

**Figure 10:**
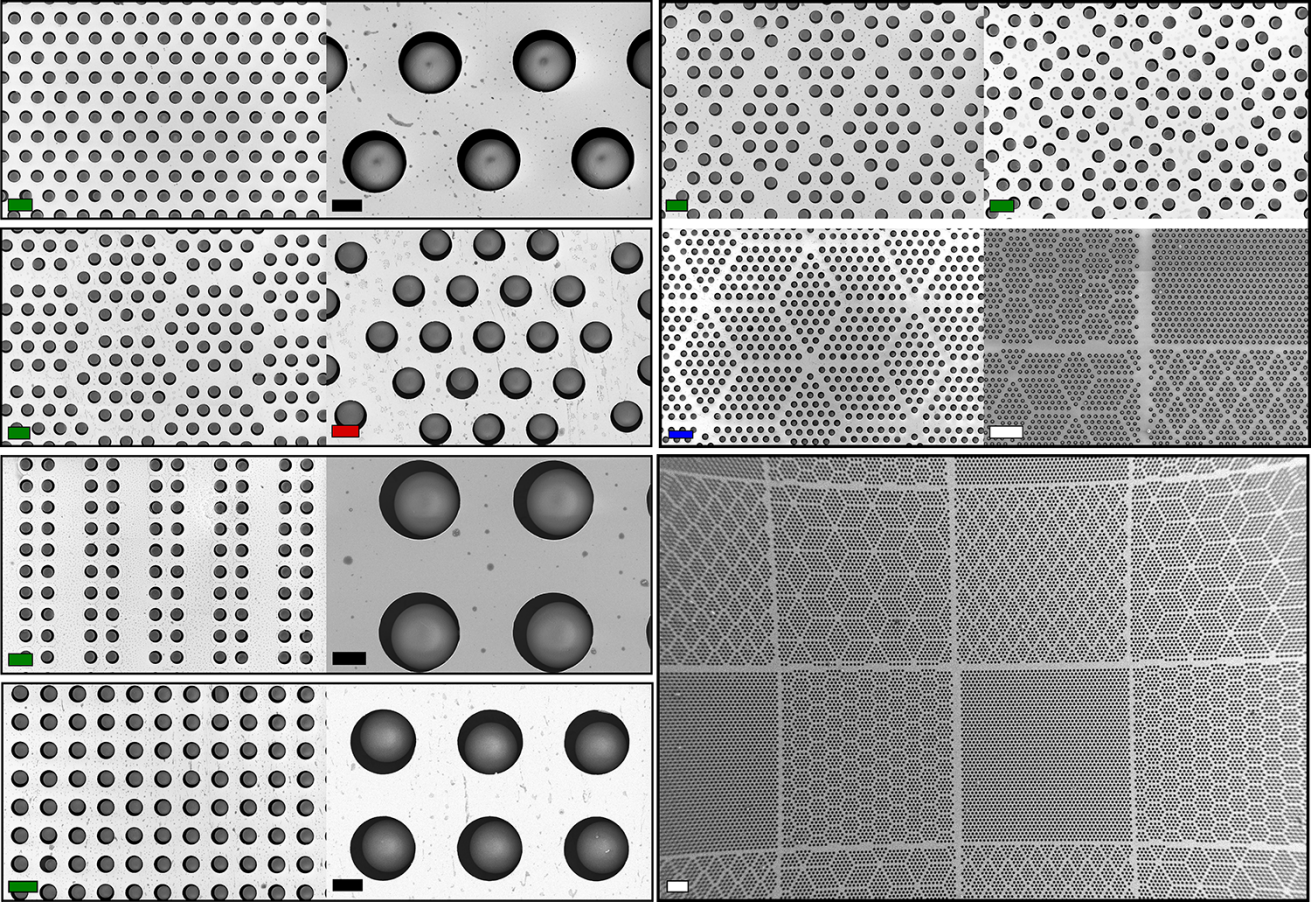
SEM pictures of particle assembly on substrates with different geometries (wet assembly, 10 μm polystyrene particles). Experimental conditions: deposition of a 30 μL drop of a 20 mg/mL suspension in ethanol to the substrate; 1 min evaporation time before initiating a unidirectional rubbing motion with a patterned PDMS substrate with 50 μm ridges. Scale bars: black = 5 μm, blue = 40 μm, green = 20 μm, red = 10 μm, and white = 100 μm.

To explore the influence of the pitch between the micromachined pockets on the filling ratio obtained via rubbing, we tested several patterns where the distance between the pockets (defined here as the pitch) was varied within a range of 1.5 (lower limit of conventional lithographic techniques) to 20 μm. These experiments were conducted to verify whether the distance along which the particles can move as a single lubrication layer along the substrates surface might influence the particle assembly. No noticeable correlation between the FR and the pitch could be detected under wet as well as dry assembly conditions: all silicon substrates tested under wet conditions all had an excellent FR of 99% or higher, regardless of the pitch (Figure 8 and Figure S6). Under dry rubbing conditions, no significant pitch effect was observed either, as now, all experiments led to a low FR below 25% (Figure 7 and Figure S6). Possibly, there might be a pitch effect for distances smaller than 1.5 μm, but this would require an alternative submicrometer patterning technique, such as e-beam lithography to produce the pocket substrates needed to test this hypothesis.^40^ To further investigate the pattern independence of the proposed wet particle assembly method, a variety of patterns were tested for 10 μm silica (Figure 9) and 10 μm polystyrene particles (Figure 10). These experiments were performed on a wafer scale on 4-inch wafers, yielding uniform filling ratios on a large scale with very little to no errors. For all the tested geometries, the particle assembly was nearly perfect (at least 99% filling ratio or higher), and the ER remained very low (<1%). This small number of remaining particles on unwanted locations generally causes difficulties for removal, as the particles are strongly adhered to the silicon substrate, often through capillary bridge formation or adhesion to an underlying particle. If desired for the application, then the very few particles that remain on top of or next to the pockets can be (partly) removed by scraping a microscope glass very slowly over the substrate. Applying a wet rubbing particle assembly followed by this simple and straightforward cleaning method, the ERs could be reduced from 1 to about 0.1%, thus practically achieving an error-free method for particle assembly on patterned silicon surfaces.

Although this technique has been developed and tested with particles in the micrometer range (5 and 10 μm), it could also possibly be employed for the assembly of submicrometer particles and even nanoparticles. For this purpose, patterned substrates with nanometer-sized pockets need to be fabricated with next-generation lithographic techniques (e.g., e-beam lithography), and this could possibly be an interesting topic for future research. We envision that the rubbing process will be able to overcome the Brownian motion of colloidal particles. However, some fine-tuning of parameters such as the evaporation time, suspension concentration, and solvent type may be required.

## Conclusions

Attempting to uniformly fill micromachined pocket arrays on silicon wafers with single particles using manual rubbing, markedly better results are obtained by working under wet conditions instead of under dry rubbing conditions. This is in contrast with similar assembly studies conducted on elastomeric substrates, where dry rubbing provides excellent results. Another difference with these studies is that the recesses in the substrate were but a fraction of the particle diameter deep, whereas in our case, the particles can completely sink into the recessed pockets. Working under wet conditions has two main advantages: first, the number of particles that is supplied to the substrate can be better controlled by means of particle suspensions, and second, van der Waals forces together with electrostatic forces are greatly reduced in a liquid environment, such that the particles move much more individually instead of in larger agglomerates. The assembly quality could also be significantly enhanced by introducing a ridge pattern on the PDMS rubbing tool, as it allows the combination of the rubbing process with the instantaneous removal of excess particles. By optimizing the direction of the pattern on the rubbing tool with respect to that of the pattern on the silicon substrate, this wet rubbing technique delivered very good assembly results on varying patterns for both silica and polystyrene particles on a wafer scale. Other parameters such as the depth of the micropockets and patterns did not seem to have a noticeable impact on the obtained assembly results.

## Supporting Information Available

Wet rubbing assembly process performed for full-size wafer substrates (MP4)
Additional experimental data (PDF)
