# Supplementary material for: Wafer-Scale Particle Assembly in Connected and Isolated Micromachined Pockets via PDMS Rubbing
Source: Langmuir. 2022 May 26;38(25):7709–19. doi: 10.1021/acs.langmuir.2c00593 (PMC9245185; doi:10.1021/acs.langmuir.2c00593)
Supplement: Supplementary file 2 — la2c00593_si_002.pdf [file la2c00593_si_002.pdf]

Supporting Information

Wafer-scale Particle Assembly in Connected and Isolated Micromachined Pockets via PDMS Rubbing

Sandrien Verloy,*,†,‡ Bert Vankeerberghen,† Ignaas S. M. Jimidar, *,†,‡ Han Gardeniers,‡ and Gert Desmet*,†

†Department of Chemical Engineering CHIS, Vrije Universiteit Brussel, Brussels, 1050, Belgium

‡Mesoscale Chemical Systems, University of Twente, Enschede, 7522 NB, The Netherlands

E-mail: s.verloy@utwente.nl; i.s.m.jimidar@utwente.nl; gedesmet@vub.be

Table of Contents

Section S1. The initial state of microspheres S2

Section S2. Wet assembly without rubbing S3

Section S3. Wet PDMS rubbing using different solvents S4

Section S4. Wet PDMS rubbing was performed at different waiting times........ S5

Section S5. Wet assembly on substrates with varied pocket depth S5

Section S6. Wet assembly on substrates with varied pitches........ S6

Section S1. The initial state of microspheres

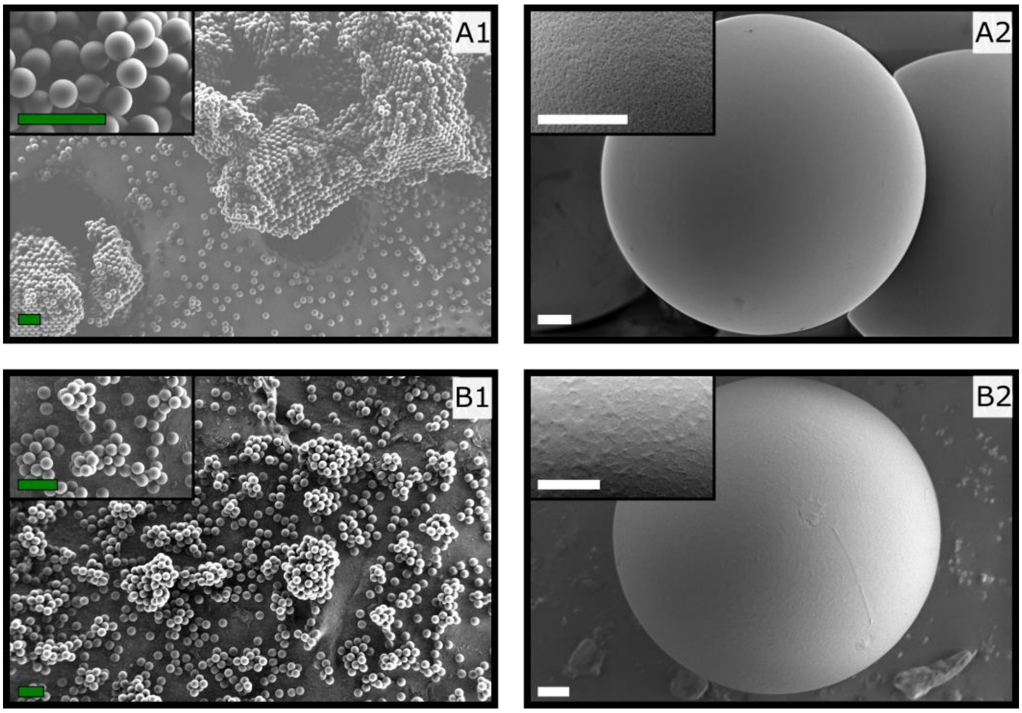

Figure S1: The silica particles in powder form, aggregated in a structure that resembles a crystal **(A1)** and a close-up of the silica particles surface **(A2)**. Polystyrene particles cluster together in smaller groups **(B1)** and present with more nano-roughness on their surface **(B2)**. Scale bar: green = 30µm, white = 1µm.

Section S2. Wet assembly without rubbing

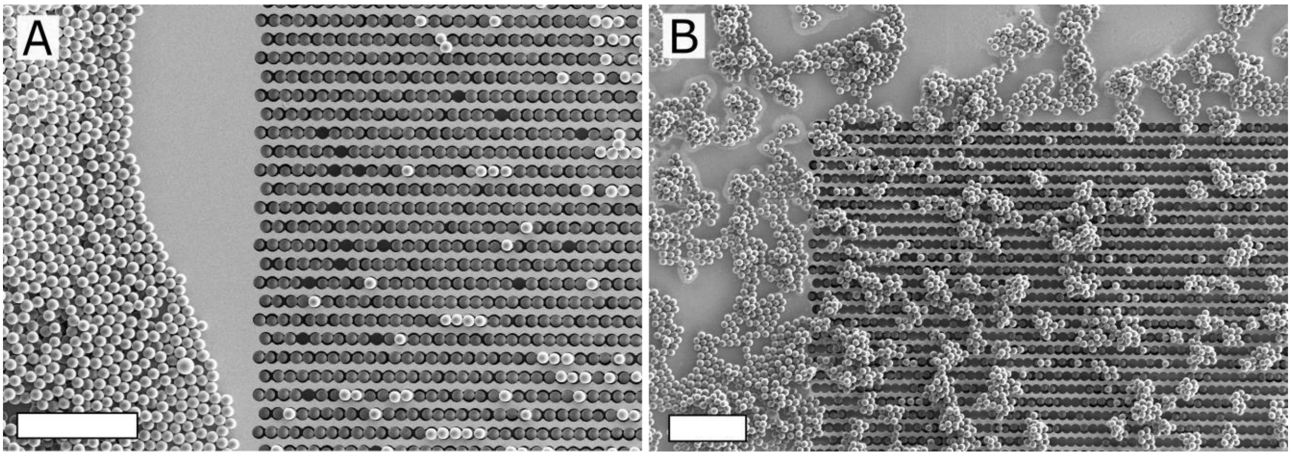

Figure S2: SEM pictures of a microgroove pattern **(A-B)** that has been filled with particles by means of evaporative driven assembly (a drop of particle suspension was supplied to the substrate and left to dry). Filling ratios were around 80% with local variations. Around the edges of the substrate, large aggregates of particles have been deposited as a result of the so called coffee ring effect. Scale bar: white = 100 µm.

Section S3. Wet PDMS rubbing using different solvents

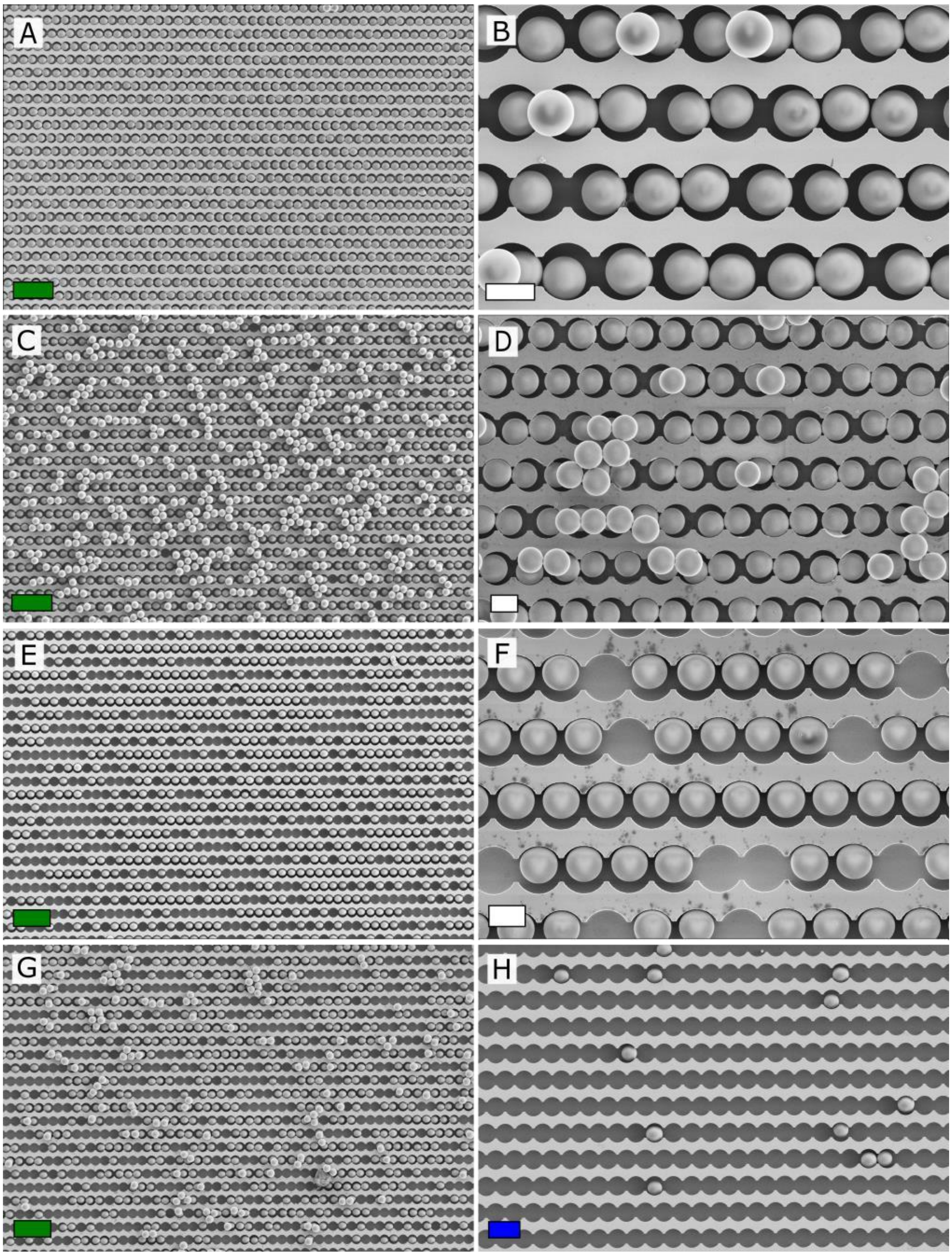

Figure S3: SEM pictures at different magnifications showing the result of several wet assembly experiments wherein different solvents were used for the particle suspensions with **(A-B)** ethanol, **(B-C)** isopropanol, **(D-E)** acetone and **(G-H)** water. The FR achieved were: >99% for ethanol (A-B), 90-99% for iso-propanol (C-D) and 30-60% with large, local variations for both acetone (E-F) and water (G-H). The ER obtained were: 0-5% for ethanol (A-B), 30-50% for propanol (C-D), <20% for acetone (E-F) and water (G-H). Experimental conditions: applied amount of particles = 30μl of a 20 mg/ml suspension in the selected solvent; 1 minute evaporation time before initiating a circular rubbing motion with a patterned PDMS substrate with 50 μm grooves. Scale bars: blue = 20 μm, green = 50 μm, white = 10 μm.

Section S4. Wet PDMS rubbing was performed at different waiting times

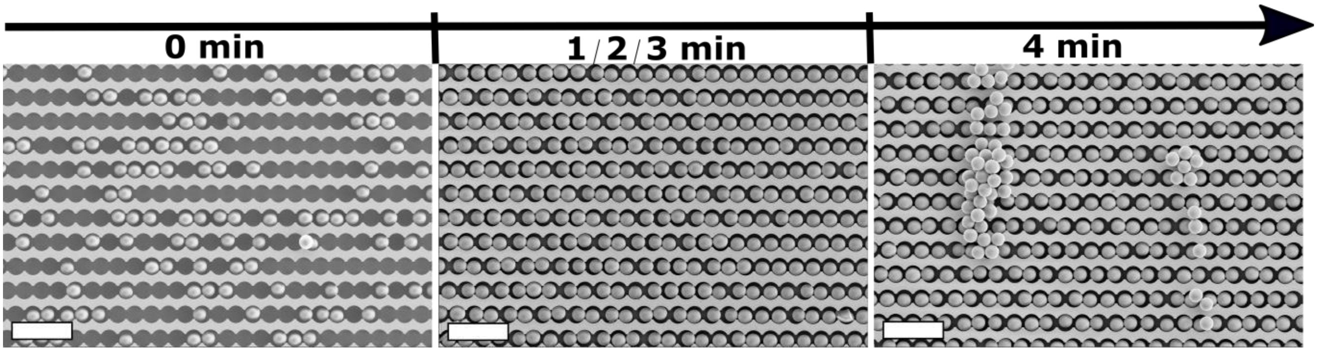

Figure S4: SEM pictures showing the result of several wet assembly experiments where the time between deposition of the suspension on the substrate and initiation of the rubbing motion was varied (suspension: 20mg/ml of 10µm silica particles suspended in ethanol; volume deposited on the substrate was 60µl). The different waiting times (in minutes) were varied between 0 and 4 minutes. The FR achieved were: 40% for 0 minutes, 100% for 1, 2 and 4 minutes. The ER achieved were: ≤1% for 0, 1 and 2 minutes and 10-15% for 4 minutes. Scale bars: white = 40 µm.

Section S5. Wet assembly on substrates with varied pocket depth

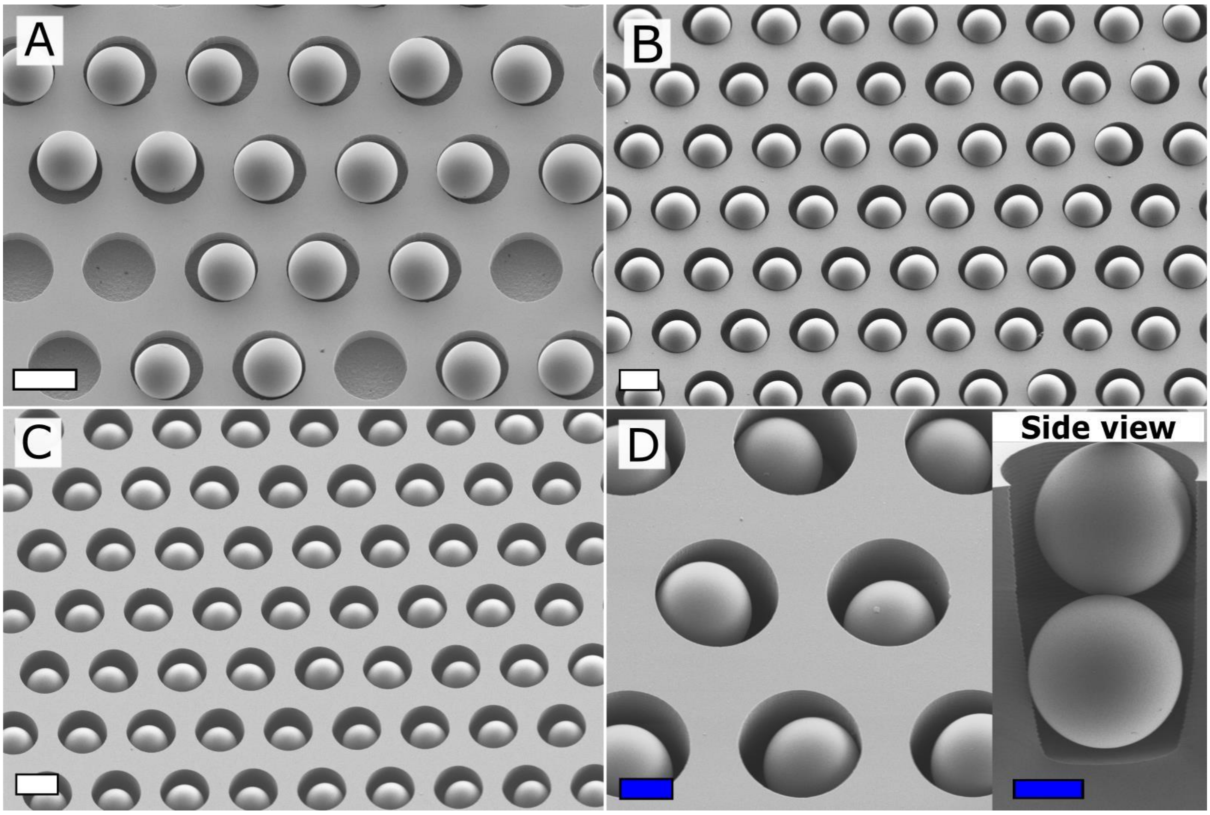

Figure S5: SEM pictures showing the result of several wet assembly experiments on separated micro-machined pockets with varying depth of the pockets (**A**: 4 µm, **B**: 8 µm, **C**: 12 µm, **D**: 24 µm). After wet assembly, samples with a depth of 4 µm were the only without satisfactory FR. Panel D shows a scenario with multiple layers of particles. Scale bar: white = 10 µm, blue = 5 µm.

Section S6. Wet assembly on substrates with varied pitches

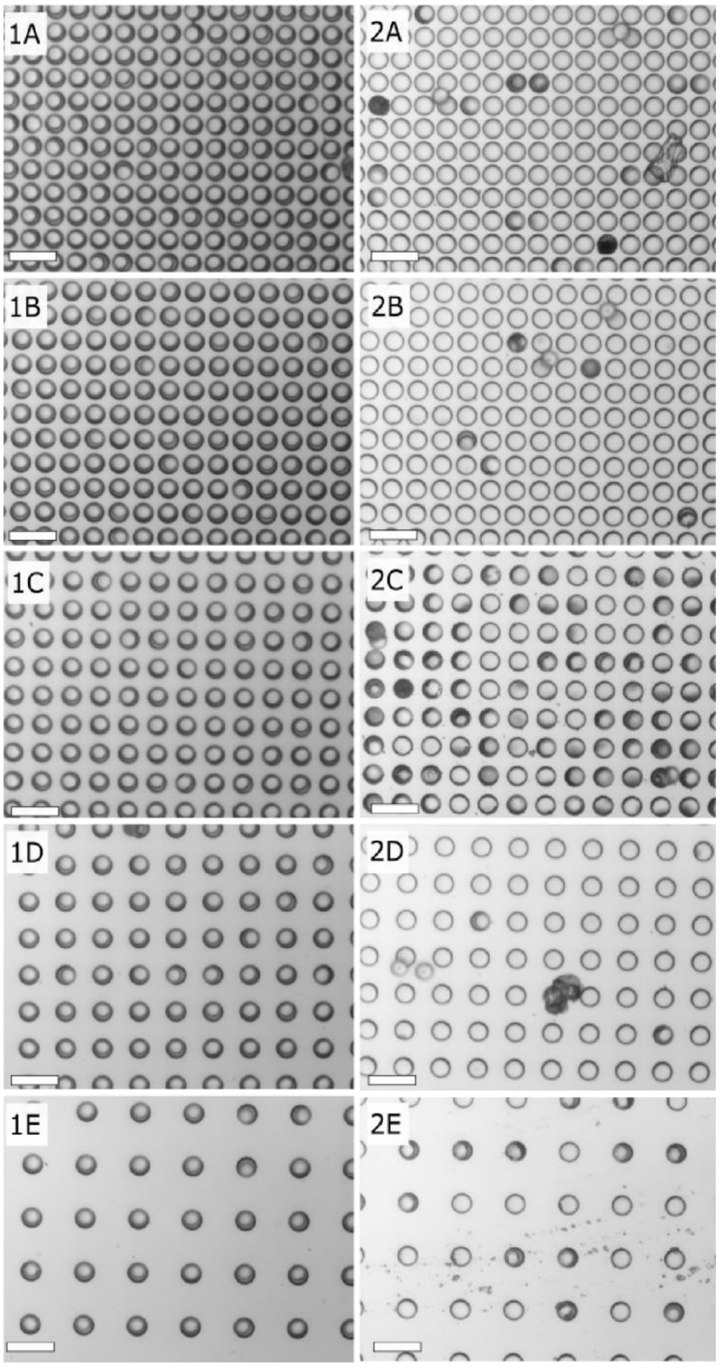

Figure S6: Optical microscope pictures showing the result of several wet **(1)** and dry **(2)** assembly experiments on separated micro-machined pockets with varying distance/pitch between them (**A**: 1,5 µm, **B**: 2,5 µm, **C**: 5 µm, **D**: 10 µm and **E**: 20 µm). After wet assembly, all samples were filled equally good with high FR (>99%) while after a dry assembly method all the samples were filled equally bad or clogged by PDMS debris. Scale bar: white = 20 µm.
